# Validation of sleep-staging accuracy for an in-home sleep electroencephalography device compared with simultaneous polysomnography in patients with obstructive sleep apnea

**DOI:** 10.1038/s41598-024-53827-1

**Published:** 2024-02-12

**Authors:** Jaehoon Seol, Shigeru Chiba, Fusae Kawana, Saki Tsumoto, Minori Masaki, Morie Tominaga, Takashi Amemiya, Akihiro Tani, Tetsuro Hiei, Hiroyuki Yoshimine, Hideaki Kondo, Masashi Yanagisawa

**Affiliations:** 1 Faculty of Health and Sports Sciences, University of Tsukuba, Tsukuba, Ibaraki 305-8574 Japan; 2 International Institute for Integrative Sleep Medicine (WPI-IIIS), University of Tsukuba, 1-2 Kasuga, Tsukuba, Ibaraki 305-8550 Japan; 3 Department of Frailty Research, Center for Gerontology and Social Science, National Center for Geriatrics and Gerontology, Obu, Aichi 474-8511 Japan; 4 Cardiovascular Respiratory Sleep Medicine, Juntendo University Graduate School of Medicine, Tokyo, 113-8421 Japan; 5 Ph.D. Program in Humanics, University of Tsukuba, Tsukuba, Ibaraki 305-8575 Japan; 6 S’UIMIN, Inc., Tokyo, 151-0061 Japan; 7 Department of Respiratory Medicine, Inoue Hospital, Nagasaki, Nagasaki 850-0045 Japan; 8 Department of General Medicine, Institute of Biomedical Sciences, Nagasaki University, 1-12-4 Sakamoto, Nagasaki, 852-8102 Japan; 9 Life Science Center for Survival Dynamics (TARA), University of Tsukuba, Ibaraki, 305-8577 Japan; 10 R&D Center for Frontiers of Mirai in Policy and Technology (F-MIRAI), University of Tsukuba, Ibaraki, 305-8575 Japan; 11 Department of Molecular Genetics, University of Texas Southwestern Medical Center, Dallas, TX 75390 USA

**Keywords:** Health care, Medical research

## Abstract

Efforts to simplify standard polysomnography (PSG) in laboratories, especially for obstructive sleep apnea (OSA), and assess its agreement with portable electroencephalogram (EEG) devices are limited. We aimed to evaluate the agreement between a portable EEG device and type I PSG in patients with OSA and examine the EEG-based arousal index’s ability to estimate apnea severity. We enrolled 77 Japanese patients with OSA who underwent simultaneous type I PSG and portable EEG monitoring. Combining pulse rate, oxygen saturation (SpO_2_), and EEG improved sleep staging accuracy. Bland–Altman plots, paired t-tests, and receiver operating characteristics curves were used to assess agreement and screening accuracy. Significant small biases were observed for total sleep time, sleep latency, awakening after falling asleep, sleep efficiency, N1, N2, and N3 rates, arousal index, and apnea indexes. All variables showed > 95% agreement in the Bland–Altman analysis, with interclass correlation coefficients of 0.761–0.982, indicating high inter-instrument validity. The EEG-based arousal index demonstrated sufficient power for screening AHI ≥ 15 and ≥ 30 and yielded promising results in predicting apnea severity. Portable EEG device showed strong agreement with type I PSG in patients with OSA. These suggest that patients with OSA may assess their condition at home.

## Introduction

Unoptimized sleep, whether qualitative or quantitative, leads to dementia, depression, cardiometabolic health issues, and increased mortality^1,2^. Approximately one billion middle-aged adults worldwide are estimated to be affected by obstructive sleep apnea (OSA) with or without symptoms^3^. An epidemiological study revealed that OSA is the most rapidly increasing form of sleep disturbance^4^.

OSA can be screened for in the laboratory and/or medical facilities using standard polysomnography (PSG) (type I PSG). This disease can also be diagnosed by monitoring oxygen saturation during sleep (e.g., 3% oxygen desaturation index [ODI], apnea–hypopnea index [AHI], and respiratory events index [REI]). However, owing to the emotional burden of an unfamiliar sleep environment, accurate sleep measurement methods in home environments to ensure precise sleep assessments need to be established^5,6^. Furthermore, while the number of patients with OSA in Japan is estimated to be over 9 million^3^, the number of PSG tests carried out annually is approximately 80,000^7^, indicating a low test-to-number of patients.

To address these limitations, several comparative validations were conducted in both laboratory and home environments using equipment such as unattended PSG (e.g., based on the American Academy of Sleep Medicine [AASM] criteria, including type II, III, and IV PSG) and wearable devices such as accelerometers and smartphone applications^5,8–12^. Type II, III, and IV involve unattended full PSG with ≥ 7, 4–7, and 1–2 channels, respectively, measuring oxygen saturation, heart rate, respiratory bands, and airflow. These measurements conducted outside the sleep laboratory using portable monitoring devices have shown substantial agreement with type I PSG^5,12,13^. However, the use of electrodes and other components in existing type II-IV PSGs remains complex, prompting the need for the development of portable electroencephalography (EEG) devices that patients can easily wear to screen for conditions such as OSA^5^. A portable EEG device that integrates electrodes, which is simpler than PSG, has been used in studies involving healthy middle-aged and/or older adults^14,15^. The advantage of this device is that although it has a smaller number of electrodes than the existing PSGs, a variety of channels can be generated by combining montages, and its accuracy improves with a smaller number of electrodes^14^ (Supplementary Fig. S1).

Accelerometers, such as actigraphs, which are easier to measure than type II–IV PSGs, exhibit high agreement with type I PSG in healthy participants for determining sleep/wake and sleep latency^8,11,16^. However, this remains an area for further investigation in patients with sleep disturbances^8,9,11^. Furthermore, the actigraph does not determine the sleep stage, and it consistently underestimates sleep parameters^5,16,17^. This inconsistency may be attributed to the high arousal response noise due to dyspnea, which leads to low accuracy^5^.

As mentioned earlier, despite the high concordance between type II-IV PSG measurements and type I PSG measurements using various instruments^12^, there are still barriers preventing patients with OSA from undergoing measurements on their own at home^12^. Therefore, we aimed to assess the level of agreement between our portable EEG device and type I PSG in patients with OSA. Additionally, it has been reported that the arousal index measured by PSG (i.e., EEG-based) can serve as a screening tool for identifying OSA^18,19^. Consequently, the process of OSA screening could be more accessible to a larger number of patients with OSA if a portable EEG device alone could effectively serve as a screening method without the need for supplementary devices. In this study, we aimed to investigate the screening capability of a portable EEG device in patients with OSA, determine the percentage of agreement with type I PSG, and evaluate the screening effectiveness using AHI thresholds of 15 and 30.

## Results

**Figure 1:**
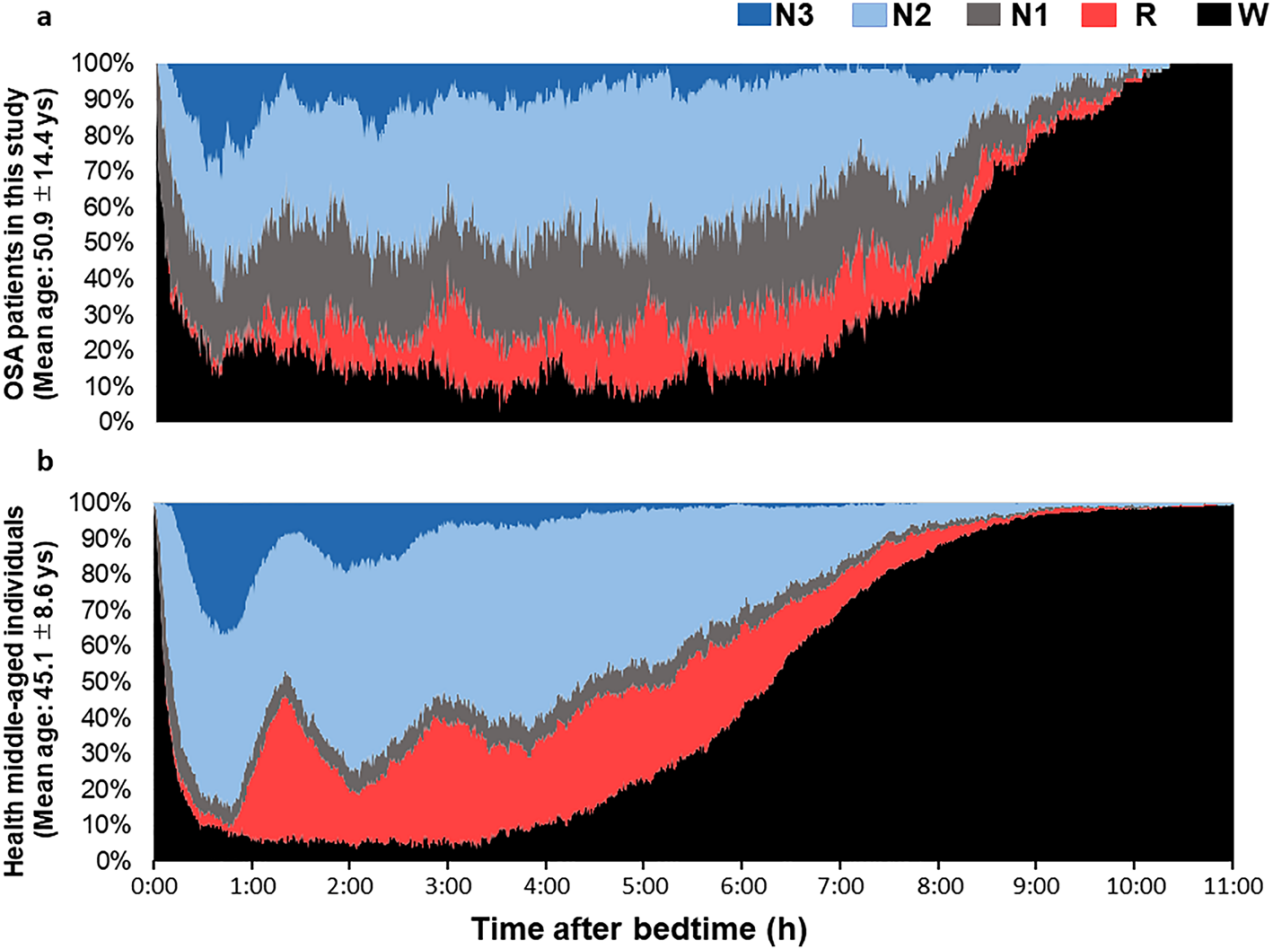
(**a**) Cumulative display of sleep architecture in all 77 patients with OSA in this study. (**b**) Cumulative display of sleep architecture in participants from a previous study who were healthy middle-aged individuals^14^. The percentage of patients in each sleep stage is shown for stage W (black), N1 (gray), N2 (light blue), N3 (blue), and R (red). W, wake; R, rapid-eye-movement sleep; N, non-REM sleep.

**Figure 2:**
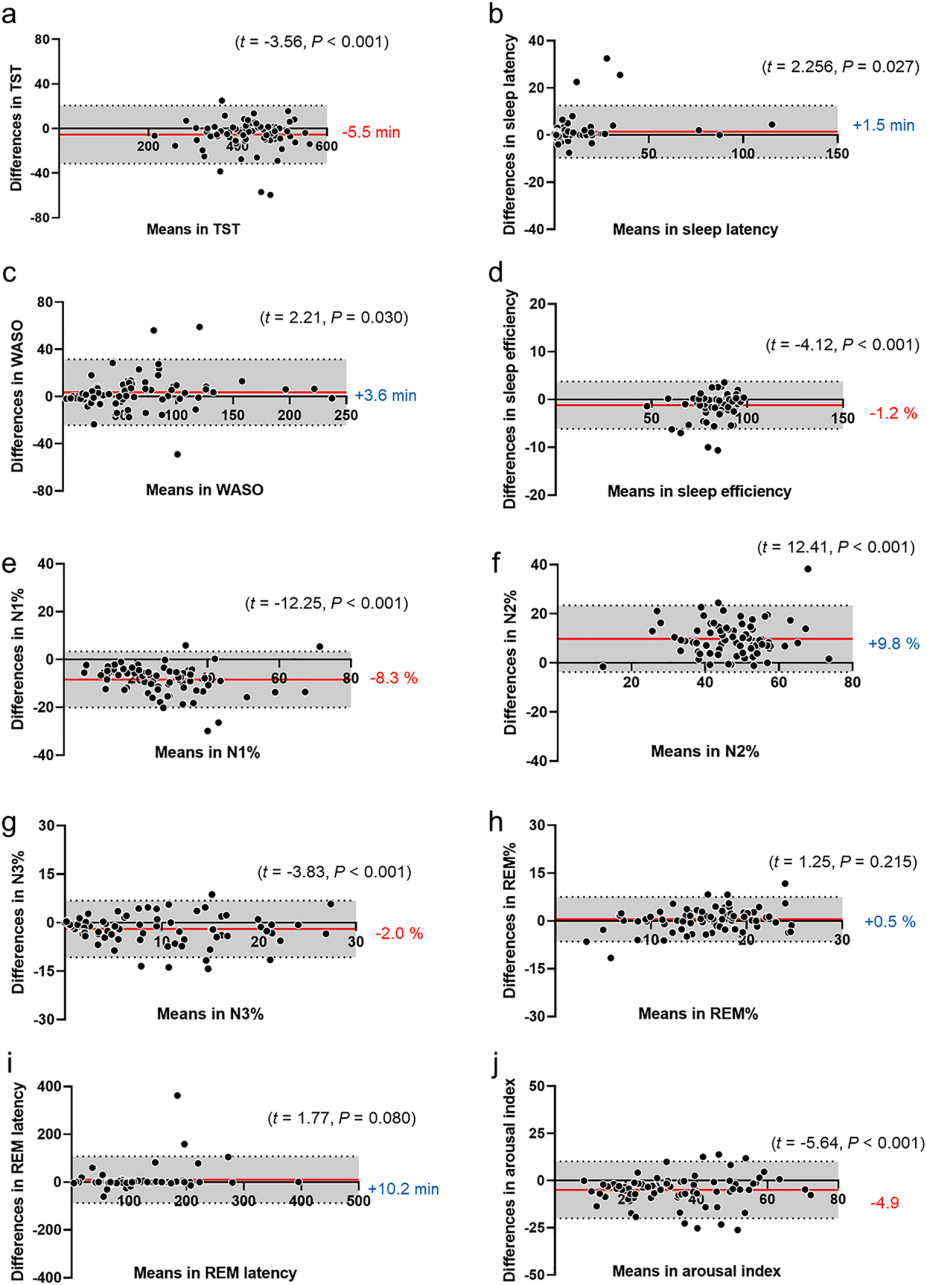
Bland–Altman plots comparing type I PSG and portable EEG device values. The Bland–Altman plots depict the mean bias (red solid line) and upper and lower limits of agreement (1.96 standard deviations from bias; black dashed lines) for each sleep parameter for the portable EEG device compared with type I PSG. The parentheses contain information about the t-test, and if the P-value is significant, it indicates that the bias, depicted to the right of the solid red line, is statistically significant. A positive solid line signifies an overestimation of the portable EEG device, while a negative line signifies an underestimation.

**Figure 3:**
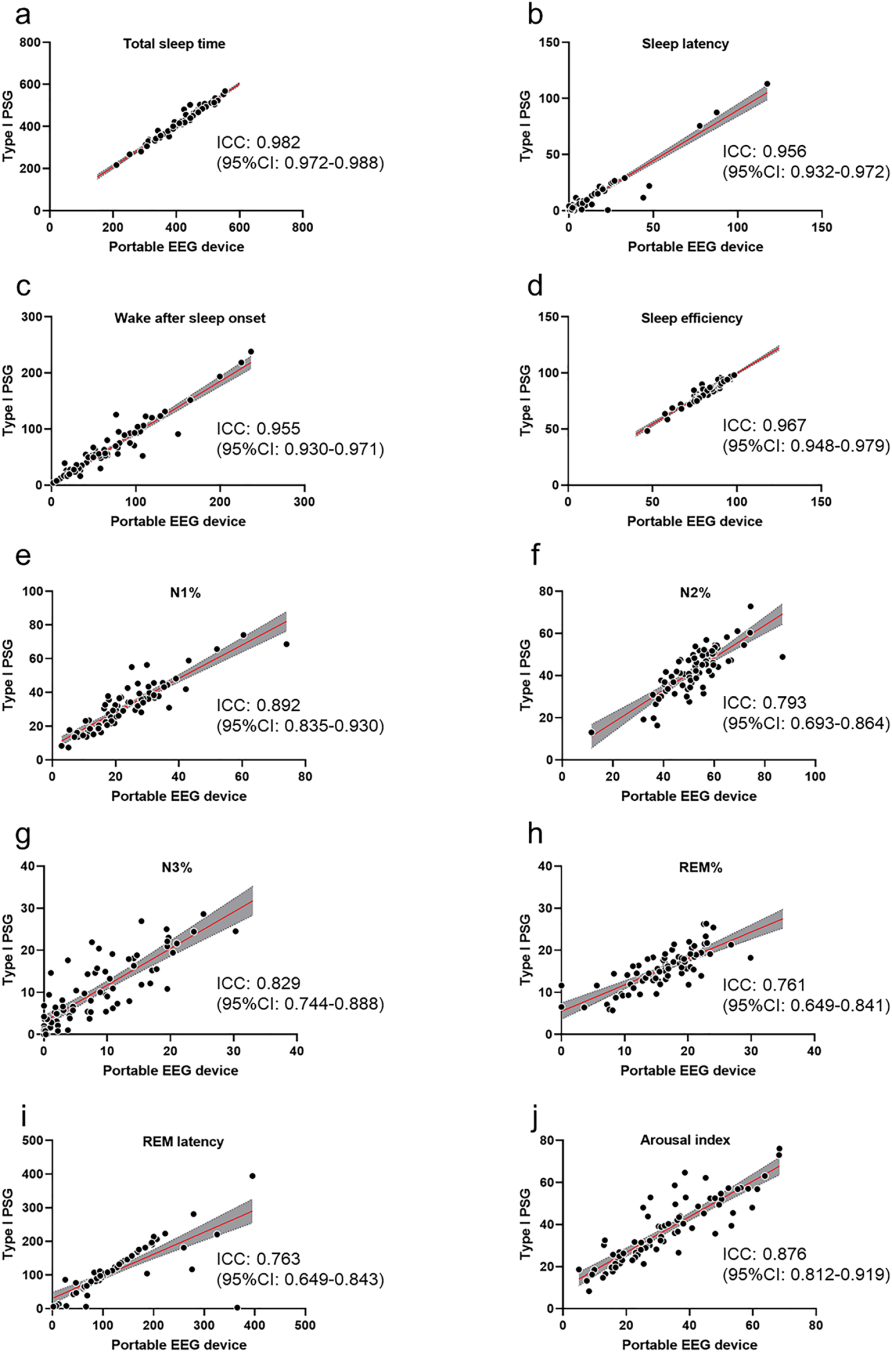
Relationship between the type I PSG and portable EEG device, showing the intraclass correlation coefficient (red solid line with 95% CI).

**Figure 4:**
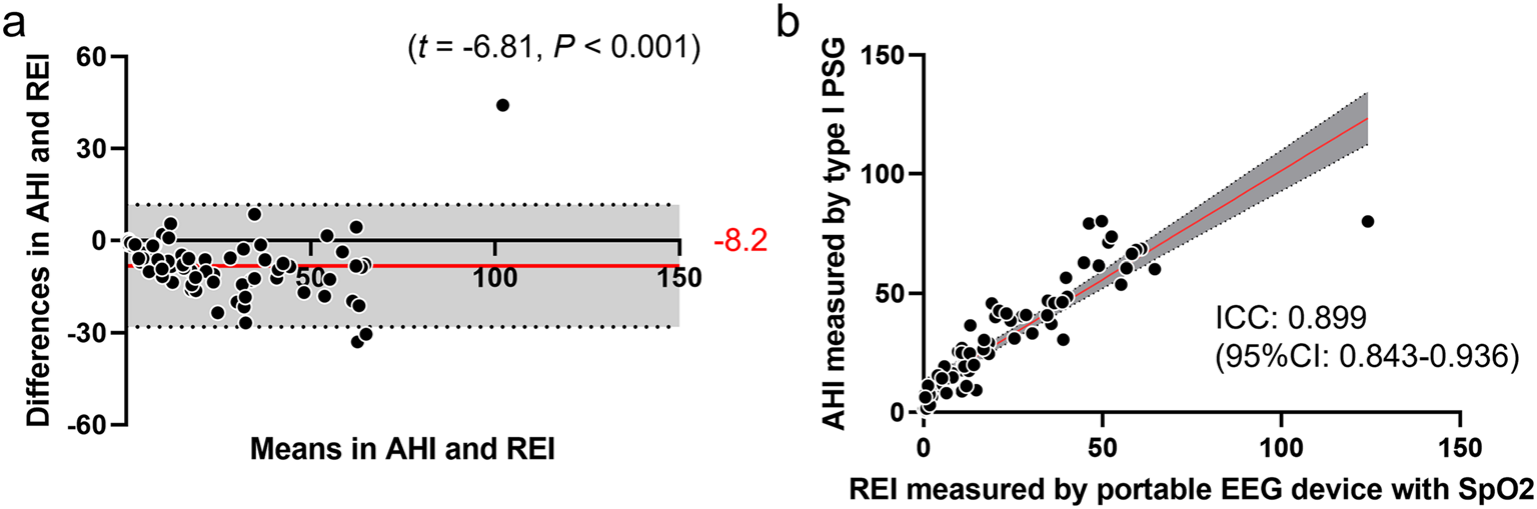
(**a**) Bland–Altman plot and (**b**) intraclass correlation coefficient was calculated between the AHI of type I PSG and the REI from portable EEG device with SpO_2_. (**a**) The Bland–Altman plots depict the mean bias (red solid line) and upper and lower limits of agreement (1.96 standard deviations from bias; black dashed lines) for each sleep parameter for the portable EEG device compared with type I PSG. The parentheses contain information about the t-test, and if the *P*-value is significant, it indicates that the bias, depicted to the right of the solid red line, is statistically significant. A positive solid line signifies an overestimation of the portable EEG device, while a negative line signifies an underestimation. (**b**) Intraclass correlation coefficient with a red solid line and 95% confidence interval.

The participant characteristics are presented in Supplementary Table S1, and the cumulative display of sleep architecture for all 77 participants is shown in Fig. 1. The average abdominal and neck circumferences were 90.4 ± 13.7 cm and 37.0 ± 4.1 cm, respectively. The total sleep time, sleep efficiency, and arousal index averaged 425.8 ± 69.5 min, 85.4 ± 9.4%, and 37.0 ± 15.1, respectively (Fig. 1 and Table 1). In this study, apnea was categorized using the AHI as mild (5–15) in 20 patients (25.9%), moderate (15–30) in 17 patients (22.1%), and severe (> 30) in 34 patients (44.1%). Six patients (7.9%) were suspected of having apnea during screening; however, they had an AHI of < 5. Table 1 presents the sleep variables measured using both the type I PSG and portable EEG devices. The results of the Bland–Altman analysis, paired t-test, and intraclass correlation coefficient (ICC) are presented in Figs. 2, 3 and 4. Significant small biases were observed in total sleep time, sleep latency, wake after sleep onset, sleep efficiency, N1, N2, and N3 rates, arousal index, and between AHI and REI (P < 0.05; Figs. 2, 4a). Notably, 95% of the values fell within the limits of agreement (LOA) for all variables, indicating a reasonable bias range of − 5.5–10.2 min and − 8.3–9.8% (Figs. 2, 4a). Furthermore, the ICC exceeded 0.75, indicating strong validity between the devices (Figs. 3, 4b).

**Table 1:** Sleep parameters measured in both devices.

	Type I PSG	Portable EEG device
	Mean ± SD	Mean ± SD
Total sleep time, min	425.8 ± 69.5	420.4 ± 69.4
Sleep onset latency, min	10.7 ± 18.4	13.5 ± 24.1
WASO, min	62 ± 46	65.6 ± 48.1
Sleep efficiency, %	85.4 ± 9.4	84.2 ± 10.1
REM latency, min	110.5 ± 67.5	118.7 ± 77.6
N1, %	31.7 ± 13.4	23.3 ± 12.1
N2, %	42.7 ± 10.5	52.5 ± 10.9
N3, %	10.0 ± 7.8	8.0 ± 7.4
REM, %	15.6 ± 4.6	16.2 ± 5.7
Arousal index, index	37.0 ± 15.1	32.0 ± 15.6
AHI and REI, index	30.7 ± 22.7	22.5 ± 22.4

*PSG* polysomnography, *WASO* wake after sleep onset, *REM* rapid eye movement sleep, *N* non-REM sleep, *AHI* apnea–hypopnea index, REI *REI* depending on SpO_2_, AHI measure by type I PSG, and REI measured by portable EEG device with SpO_2_.

The confusion matrix for the five sleep stages (Wake, N1, N2, N3, REM) out of a total of 75,677 epochs (Table 2). The agreement between the two devices was 10,032 (91.7%), 11,380 (55.8%), 24,921 (90.0%), 4,230 (66.6%), and 8,772 (85.2%) epochs for Wake, N1, N2, N3, and REM, respectively (Table 2). The overall kappa coefficient was 0.708.

**Table 2:**
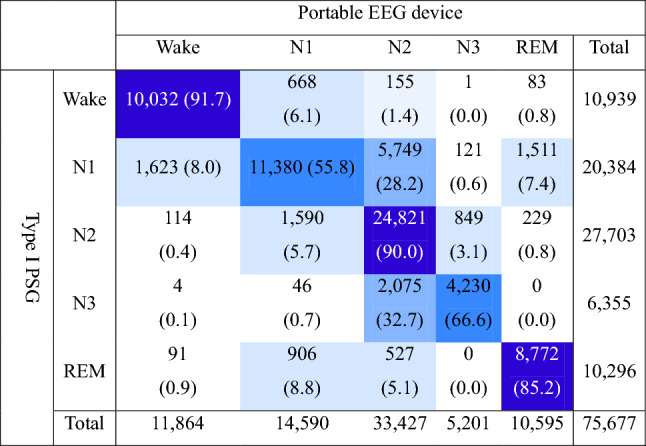
Confusion matrix for sleep stage classification in all epochs of all recordings (n = 75,677).

Parentheses indicate percentage agreement between two devices.

*PSG* polysomnography, *REM* rapid eye movement sleep, *N* non-REM sleep.

**Figure 5:**
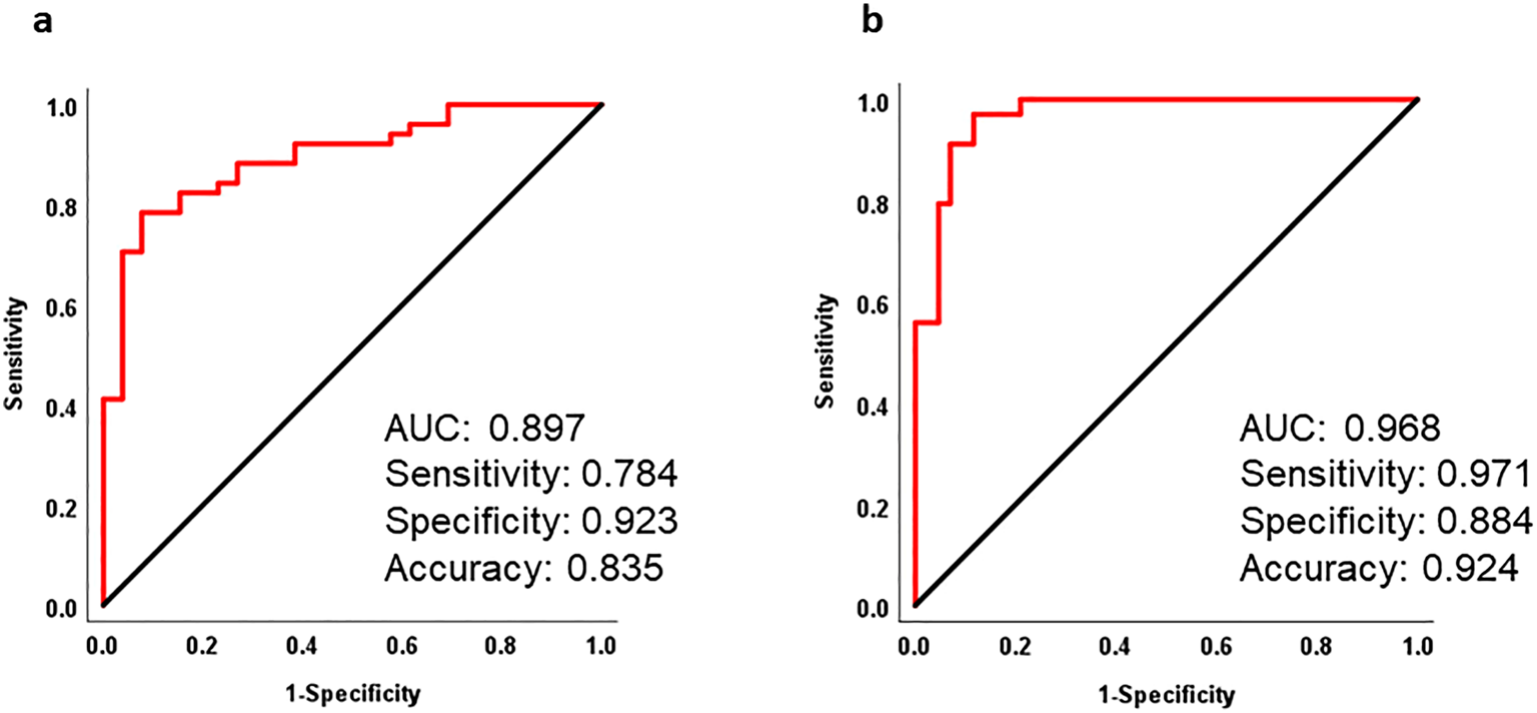
ROC curves for OSA diagnosis using the arousal index measured using the portable EEG device. (**a**) ROC curve for OSA diagnosis using the arousal index at an AHI threshold of ≥ 15 events/h. (**b**) ROC curve for OSA diagnosis using the arousal index at an AHI threshold of ≥ 30 events/h. OSA, obstructive sleep apnea. Both (**a**) and (**b**) ROC curves adjusted for sex, age, and BMI.

The epoch-by-epoch analysis showed good accuracy in identifying sleep stages between the two devices: Wake (96.4), N1 (83.9), N2 (85.0), N3 (95.8), and REM (95.6) (Table 3). Furthermore, the specificity of the sleep stages was significant: Wake (97.1), N1 (93.8), N2 (82.2), N3 (98.6), and REM (97.3). The sensitivities for Wake (88.2), N2 (88.6), and REM (84.1) were higher than those of N1 (54.3) and N3 (53.9) (Table 3). The trend of high concordance for arousal, N2, and REM, while relatively low for N1 and N3, remained consistent even when the AHI levels were different. (Supplementary Table S2, Supplementary Figs. S2 and S3). Notably, N1 and N2 PABAKs showed reductions of 18.4% and 8.2%, respectively, in cases where AHI was ≥ 30 when compared with AHI < 15 (Supplementary Fig. S3). The arousal index obtained from the portable EEG device had an area under the curve (AUC) of 0.897, with sensitivity, specificity, and accuracy for AHI ≥ 15 being 0.784, 0.923, and 0.835, respectively. Additionally, the AUC, sensitivity, specificity, and accuracy for AHI ≥ 30 were 0.968, 0.971, 0.971, and 0.924, respectively (Fig. 5).

**Table 3:** Overall epoch-by-epoch analysis for type I PSG and portable EEG device.

	Accuracy	Sensitivity	Specificity	PABAK
Wake	96.4 (2.8)	88.2 (13.7)	97.1 (2.9)	0.93 (0.1)
N1	83.9 (6.2)	54.3 (13.1)	93.8 (4.2)	0.68 (0.1)
N2	85.0 (5.5)	88.6 (8.5)	82.2 (7.3)	0.70 (0.1)
N3	95.8 (3.0)	53.9 (33.7)	98.6 (1.8)	0.92 (0.1)
REM	95.6 (2.1)	84.1 (15.5)	97.3 (2.1)	0.91 (0.1)

Standard errors are in parentheses.

*PSG* polysomnography, *REM* rapid eye movement sleep, *N* non-REM sleep, *PABAK* prevalence and bias-adjusted kappa.

## Discussion

This study is one of the few to confirm the concordance of portable EEG devices with type I PSG in patients with OSA^20^. N1 and N3 exhibited lower sensitivities than Wake, N2, and REM (Table 3). To examine the inter-rater variability of sleep staging, 10 skilled EEG analysts analyzed EEG data from 70 patients in the same sleep period, with results consistent with those of previous studies. Specifically, there was poor agreement for N1 and N3^21^. Considering that the pattern of N1 decreased with the increasing severity of apneic symptoms (Supplementary Fig. S2), it might be necessary for future studies to further refine the EEG criteria for patients with apnea. A previous study^22^ investigating the agreement between portable single-channel EEG devices and type I PSG in relatively healthy middle-aged and elderly persons (without apneic symptoms) revealed similar findings, although the overall accuracy, specificity, and sensitivity were lower than those observed in the present study^22^. Notably, the N1 value of 20.6 is low^22^. Referring to the confusion matrix results (Table 2), there was a reduction in sensitivity for determining N1 and N3 compared to the other stages (Table 3). The portable EEG device frequently misjudged type I PSG’s N1 as N2 (28.2%) and N3 as N2 (32.7%).

The reasons for this misjudgment could not be determined in this study; however, there are three possibilities. First, as with the portable single-channel EEG device^21^, the use of fewer channels than in type I PSG may have contributed to a decrease in sensitivity for N1, which requires more detailed assessments^23^. Indeed, concerning forehead EEG devices, as emphasized in previous studies, sweat artifacts, which manifest as high-amplitude and low-frequency waves, are associated with the challenge of observing the N2/N3 transition without an epoch delay, causing a decrease in sensitivity^24^. Second, patients with OSA experience more noise than healthy subjects^25^, which could lead to the misclassification of N1 as Wake or REM or N2 as N3, even when analyzed by a sleep EEG analyst. This shows that the interprofessional agreement in EEG analysis was 82.0%, which is consistent with that of a previous study^26^. Although the sensitivity of N1 and N3 in determining sleep stage was low, the agreement was generally high; hence, there is a possibility of home measurements in the future with a portable EEG device. Third, drawing on our earlier studies^14,15^, patients with OSA exhibited reduced N2 sleep and increased N1 sleep (there might have been a misinterpretation of N1 and N2) (Fig. 1a and b), even when their AHI was not greater than 100^27^. Since inter-rater reliability is lowest for N1 determination^28^, it is reasonable that N1 determination performance would decrease with the severity of OSA in patients with increased N1.

To reduce the burden of OSA, various devices have been developed, including wearable devices (e.g., portable EEG devices and/or actigraphy) and smartphone applications^5,17^. Although numerous studies on healthy subjects have demonstrated results comparable to those of Type I PSG, only a limited number of studies have been conducted on patients with OSA^16^. In healthy subjects, there is high agreement between wearable devices and PSG^5^. However, it has been reported that assessing sleep onset latency is challenging in patients with OSA, although some studies have incorporated various algorithms to enhance accuracy^29–31^.

Furthermore, this study demonstrated that the arousal index, calculated from a portable EEG device, can effectively screen for moderate (AHI ≥ 15) and severe (AHI ≥ 30) apnea. Notably, screening efficacy was higher when using AHI ≥ 30 as the criterion than AHI ≥ 15, as shown in Fig. 4. Our results confirmed that the AHI cut-off values of 15 and 30 or higher are an EEG-based arousal index ≥ 25 and ≥ 32, respectively. While previous studies have reported that the arousal index of type I PSG can be used to screen for moderate (AHI ≥ 15) and severe (AHI ≥ 30) apnea^18,19^, results from the portable EEG device align in a manner that enables screening with comparable efficacy.

The use of portable EEG device systems has become increasingly institutionalized. Meta-analyses have reported respiratory event measurements (e.g., AHI, ODI, respiratory disturbance index, oxygen saturation, and lowest oxyhemoglobin saturation) from both type I PSG and portable EEG devices conducted at home to be within the range of 0.832–0.942^32^. This study further suggests that the sleep EEG of patients with apnea can be accurately assessed using a portable EEG device. Recent advancements in portable EEG devices, which are easier to use than type II–IV PSG, are expected to lead to further developments in the future. The portable EEG device used in this study uploads the EDF data to the cloud as soon as the overnight measurement is complete. Users can promptly review the previous night’s sleep results upon waking up, utilizing machine learning and AI to determine their sleep stages^33,34^. In this study, a professional EEG analyst performed all assessments to ensure reliability. However, portable PSGs equipped with AI analysis capabilities are expected to advance further.

Our study had a few limitations. Previous studies have proposed integrating various portable devices that can be easily measured at home to improve measurement accuracy^21^. We also performed sleep staging by combining SpO_2_ and pulse rate obtained with type I PSG on a portable EEG device and found a high concordance rate. However, further studies are required to determine a simple method for recording respiratory functions and heart rate variability on a portable EEG device. Second, to maintain natural sleep patterns, no restrictions were placed on sleep duration, resulting in varying total sleep times among the participants. Sleep stages were quantified as percentages relative to total sleep time. It is important to mention that participants were required to sleep at a medical institution’s sleep laboratory to enable a direct comparison with type I PSG. Therefore, future studies are required to validate these findings in the home environment. Third, while this study demonstrated results comparable to those of type I PSG in screening for an AHI of 15 or 30 using the arousal index measured by a portable EEG device, it remains unclear whether it can be used to screen healthy individuals. This is because the study population included patients with apnea, suggesting that further research is required in this area. Finally, out of a total of 90 measurements taken with the portable EEG device, there were 11 cases (12%) of instrumental problems, including electrode disconnection and measurement time deviation. Failure rates ranging from 3–18% for measurements obtained solely with a portable EEG device have been reported^35^, which is considered a reasonable failure rate given the study’s simultaneous measurement of type I PSG and the portable EEG device. Additionally, future studies should examine the inter-rater validity of test–retest reliability.

## Conclusions

In patients with OSA, our portable EEG device demonstrated good agreement with type I PSG, although more work is required to improve the agreement for N1 and N3 screening. Measurements could be conducted in a home setting, significantly reducing the burden on patients with OSA. Furthermore, the EEG-based arousal index, which predicts the degree of apnea without the need for a separate device to measure respiratory events, showed good results even with the portable EEG device in this study. This suggests that patients with OSA may be able to comfortably use this device in their homes to determine their degree of apnea.

## Methods

### Study design and participants

This cross-sectional study was conducted between August 2021 and March 2022. A total of 90 patients with OSA were recruited from the Sleep Center of Shunkaikai Inoue Hospital, Nagasaki, Japan. The inclusion criteria were age > 20 years and suspected of having an OSA with an AHI measurement ≥ 5 or a 3% ODI of ≥ 5 on an at-home test with a wearable device or a simple pulse oximeter. Instrumental problems, including measurement time deviations, were experienced with Type I PSGs (n = 2) and portable EEG devices (n = 5). Additionally, portable EEG devices with electrode-dislodgement measurement failures (n = 6) were excluded from the analysis. Finally, 77 patients with OSA were included in the analysis. Participants with suspected sleep apnea wore both type I PSG and portable EEG devices simultaneously and slept and woke up at their usual time. As soon as the patient went to bed, the lights were turned off, and the Type I PSG and portable EEG device were started. Similarly, as soon as the patient woke up, the lights were turned on, and the recording from both devices was stopped. In addition, sleep staging calculations were performed after matching the time of light on/off on both devices.

This study was performed in accordance with the principles of the Declaration of Helsinki, and all experimental protocols were approved by the Ethics Committee of the Japan Medical Association (Ref. 2014-4). Additionally, all participants provided informed consent.

### Measurements

#### Standards polysomnography (type I PSG)

Type I PSG was recorded using Alice 6 LDE (Philips Respironics, PA, USA). The recording system of this device consisted of six EEG electrode sites (F3–M2, F4–M1, C3–M2, C4–M1, O1–M2, and O2–M1). Two electrooculograms and one submental electromyogram with pulse rate and SpO_2_ were adopted and recorded during sleep. The records were scored every 30 s to classify the sleep stages as awake (stage W), non-rapid eye movement (REM) (stage N), N1, N2, N3, and REM. We calculated the percentage of total sleep time for each sleep stage. Measurements during sleep onset latency were classified as stage W. REM latency was measured until the first R stage, which appeared after sleep onset. Sleep efficiency was calculated as the sum of N1, N2, N3, and REM sleep (i.e., total sleep time) divided by the total time spent in bed, multiplied by 100. Wake after sleep onset (WASO) was defined according to standard AASM criteria^36^. Additionally, we calculated the arousal index during the sleep period as follows: the number of arousals on EEG divided by the total sleep time (h). The AHI was calculated by assessing the frequency of apneas and hypopneas based on oxygen saturation, nasal respiratory flow sensor readings, and respiratory capacity determined by thoracoabdominal movement per total sleep time measured using type I PSG. To assess whether the arousal index measured from each EEG could function as a screening tool for AHI, we categorized the AHI into moderate apnea (AHI ≥ 15) and severe apnea (AHI ≥ 30)^10^.

#### Portable EEG device

A portable EEG was recorded using the Insomnograf K2 (S’UIMIN Inc., Tokyo, Japan), which is lightweight (162 g) and easily attached and detached because of the soft-sticking integrated electrodes (Supplementary Fig. S1). In healthy subjects, it demonstrated 86.9% agreement with type I PSG and a kappa coefficient of 0.80^14,15^. The recording system of this device consisted of four EEG electrodes (Fp1, Fp2, M1, and M2) and one reference electrode (Fpz), according to the 10–20 system. The montage was combined with four electroencephalogram derivations (Fp1–M2, Fp2–M1, Fp1–average M, and Fp2–average M), using Fp1–Fp2 and Fp2–Fp1 for left and right electrooculography, respectively, and M1–M2 for chin electromyography to analyze sleep staging. To enhance the accuracy of sleep staging based on respiratory events, we integrated the pulse rate and SpO_2_ data from a type I PSG with EEG data from a portable EEG device for sleep-staging determination^37^. However, the portable EEG device, pulse, and SpO_2_ data sheets were blinded as a separate set from the type I PSG. We calculated the REI depending on SpO_2_ (REI) (i.e., it shares the same concept as AHI) by counting the total number of respiratory events based on oxygen saturation, pulse rate, and EEG-detected respiratory events and dividing by the total sleep time recorded by the portable EEG device. To classify the sleep stages, recordings were made every 30 s, and criteria similar to the type I PSG method described above were used^36^. Sleep staging for type I PSG and portable EEG devices was performed by the same sleep EEG analyst with 40 years of working experience. The portable EEG and type I PSG EDF were used to determine sleep staging by a sleep EEG analyst who had no visibility of the respective results. A third-party researcher linked both datasheets.

### Potential confounders

To identify potential confounders, we included continuous variables such as age, body mass index (BMI), and abdominal and neck circumference (continuous variable). Categorical variables (“yes” or “no”), including sex (“male” or “female”), tobacco-smoking status (“current” or “previous/never”), alcohol consumption (“drinker” or “non-drinker”), and medical history of hypertension, diabetes, dyslipidemia, and hyperuricemia, were evaluated. Furthermore, depressive symptoms were assessed using the Center for Epidemiologic Studies Depression Scale^38^. Finally, subjective sleep parameters were measured using the Epworth Sleepiness Scale^39^ and the Athens Insomnia Scale^40^.

### Statistical analysis

Bland–Altman analysis was used to compare each device, illustrating individual night differences compared to type I PSG and portable EEG devices. This analysis also presented the overall levels of bias and the LOA. A statistically significant paired t-test was used to assess bias. Furthermore, the ICC and 95% confidence interval (CI) were calculated for both devices. ICC values > 0.75, 0.40–0.75, and < 0.40 were very good, fair to good, and poor, respectively^41^. Additionally, an epoch-by-epoch analysis was conducted across all 30 s epochs for each device. This analysis involved calculating the accuracy, sensitivity, specificity, prevalence, and bias-adjusted kappa (PABAK) by referring to prior studies^42^.

Receiver Operating Characteristic (ROC) curve analysis was performed to investigate the potential of the arousal index in screening for apnea. This analysis utilized moderate (AHI ≥ 15) and severe (AHI ≥ 30) apnea as dependent variables while adjusting for independent variables such as sex, age, BMI, and arousal index measured with a portable EEG device. The AUC, accuracy, sensitivity, and specificity were calculated.

Bland–Altman and epoch-by-epoch analyses were conducted using R software (R Foundation for Statistical Computing, Vienna, Austria), and the ICC and ROC curve analyses were conducted using SPSS version 26.0 (IBM Corporation, Armonk, NY, USA). A two-tailed statistical significance was set at *P* < 0.05 (two-tailed).

### Supplementary Information

Supplementary Information.

## Data Availability

The data used in this study was licensed to S’UIMIN, Inc. The data have not been made publicly available and can be used in the future for the development of medical devices and diagnostic technologies. Proposals and requests for data access should be emailed to the corresponding authors.
