# Supplementary material for: Validation of sleep-staging accuracy for an in-home sleep electroencephalography device compared with simultaneous polysomnography in patients with obstructive sleep apnea
Source: Sci Rep. 2024 Feb 12;14:3533. doi: 10.1038/s41598-024-53827-1 (PMC10861536; doi:10.1038/s41598-024-53827-1)
Supplement: Supplementary file 1 — Supplementary Information. [file 41598_2024_53827_MOESM1_ESM.docx]

**Supplementary Information**

Supplementary Information for **Validation of sleep-staging accuracy for an in-home sleep electroencephalography device compared with simultaneous polysomnography in patients with obstructive sleep apnea**

Jaehoon Seol, Shigeru Chiba, Fusae Kawana, Saki Tsumoto, Minori Masaki, Morie Tominaga, Takashi Amemiya, Akihiro Tani, Tetsuro Hiei, Hiroyuki Yoshimine, Hideaki Kondo, and Masashi Yanagisawa

Correspondence should be addressed to Jaehoon Seol

Email: [seol.jaehoon.ge@u.tsukuba.ac.jp](mailto:seol.jaehoon.ge@u.tsukuba.ac.jp)

or

Masashi Yanagisawa

Email: yanagisawa.masa.fu@u.tsukuba.ac.jp

**This PDF file includes:**

Table S1 and S2

Fig. S1 to S3

**Supplementary Table S1. Participant characteristics (n = 77)**

|  |  | Mean | ± | SD | Min | Max |
| --- | --- | --- | --- | --- | --- | --- |
| Age (years) | | 51.4 | ± | 14.4 | 23 | 81 |
| Female, n (%) | | 19 | (24.7) | |  |  |
| Body mass index, kg/m^2^ | | 25.8 | ± | 5.0 | 16.6 | 43.9 |
| Abdominal circumference, cm | | 90.4 | ± | 13.7 | 60.5 | 126.1 |
| Neck circumference, cm | | 37.0 | ± | 4.1 | 29.0 | 49.5 |
| Smoking history, n (%) | | 33 | (42.9) | |  |  |
| Alcohol consumption (drinker), n (%) | | 25 | (32.5) | |  |  |
| **AHI, index** | | 30.7 | ± | 23.3 | 0.5 | 80.7 |
| Mild OSA (5 to 15 events/hour) | | 20 | (25.9) | |  |  |
| Moderate OSA (15 to 30 events/hour) | | 17 | (22.1) | |  |  |
| Severe OSA (≥ 30 events/hour) | | 34 | (44.1) | |  |  |
| **Medical history**, n (%) | |  |  | |  |  |
| Hypertension | | 42 | (54.5) | |  |  |
| Diabetes | | 12 | (15.6) | |  |  |
| Dyslipidemia | | 39 | (50.6) | |  |  |
| Hyperuricemia | | 17 | (22.1) | |  |  |
| **CES-D score, pts** | | 10.3 | ± | 7.5 | 0 | 34 |
| Depression (≥ 16 pts), n (%) | | 23 | (29.9) | |  |  |
| **ESS score, pts** | | 8.0 | ± | 5.0 | 0 | 22 |
| Excessive daytime sleepiness (≥ 11 pts), n (%) | | 18 | (23.4) | |  |  |
| **AIS score, pts** | | 4.3 | ± | 3.1 | 0 | 15 |
| Insomnia (≥ 6 pts) | | 23 | (29.9) | |  |  |

AHI, apnea-hypopnea index; OSA, obstructive sleep apnea; CES-D, Center for Epidemiologic Studies Depression Scale; ESS, Epworth Sleepiness Scale; AIS, Athens Insomnia Scale.

**Supplementary Table S2.** Bland-Altman analysis, paired t-tests, and ICC by apnea severity

|  | Bland-Altman analysis,  mean (-1.96SD, +1.96SD) | | | Paired *t* test,  *t* (*p* value) | | | ICC,  coefficient (95%CI) | | |
| --- | --- | --- | --- | --- | --- | --- | --- | --- | --- |
|  | AHI | | | AHI | | | AHI | | |
|  | <15 | 15-30 | >30 | <15 | 15-30 | >30 | <15 | 15-30 | >30 |
|  | n = 25 | n = 17 | n = 35 | n = 25 | n = 17 | n = 35 | n = 25 | n = 17 | n = 35 |
| Total sleep time, min | -4.9  (-30.2, 20.4) | -9.3  (-43.8, 25.2) | -4.2  (-25.8, 17.3) | -1.91  (0.069) | -2.18  (0.045) | -2.25  (0.032) | 0.98  (0.96, 0.99) | 0.97  (0.91, 0.99) | 0.99  (0.98, 0.99) |
| Sleep latency, min | 0.7  (-4.8, 6.1) | 1.9  (-13.8, 17.5) | 1.9  (-9.8, 13.5) | 1.18  (0.248) | 0.96  (0.353) | 1.82  (0.077) | 0.99  (0.98, 0.99) | 0.91  (0.78, 0.97) | 0.93  (0.87, 0.97) |
| WASO, min | 4.3  (-21.5, 30.0) | 8.1  (-27.2, 23.4) | 2.4  (-16.0, 20.8) | 1.62  (0.117) | 1.85  (0.083) | 1.49  (0.147) | 0.92  (0.84, 0.97) | 0.84  (0.61, 0.94) | 0.99  (0.98, 0.99) |
| Sleep efficiency, % | -1.0  (-5.9, 3.9) | -1.8  (-7.8, 4.2) | -0.9  (-5.1, 3.4) | -2.02  (0.054) | -2.42  (0.028) | -2.36  (0.024) | 0.95  (0.89, 0.98) | 0.92  (0.79, 0.97) | 0.99  (0.63, 0.99) |
| N1, % | -6.4  (-14.4, 1.6) | -7.8  (-21.2, 5.6) | -9.9  (-22.5, 2.7) | -7.85  (<0.001) | -4.70  (<0.001) | -9.01  (<0.001) | 0.89  (0.77, 0.95) | 0.73  (0.39, 0.89) | 0.87  (0.76, 0.93) |
| N2, % | 8.2  (-4.1, 20.6) | 9.1  (-1.3, 19.4) | 10.5  (-2.1, 23.0) | 6.52  (<0.001) | 7.10  (<0.001) | 9.53  (<0.001) | 0.77  (0.56, 0.89) | 0.83  (0.59, 0.93) | 0.80  (0.64, 0.89) |
| N3, % | -2.3  (-11.2, 6.6) | -2.2  (-9.0, 4.5) | -1.2  (-10.0, 7.6) | -2.50  (0.020) | -2.67  (0.017) | -1.60  (0.120) | 0.85  (0.69, 0.93) | 0.90  (0.74, 0.96) | 0.90  (0.74, 0.96) |
| REM, % | 0.4  (-6.2, 7.1) | 1.0  (-7.3, 9.2) | 0.7  (-4.8, 6.2) | 0.60  (0.553) | 0.95  (0.355) | 1.47  (0.150) | 0.84  (0.68, 0.93) | 0.46  (-0.01, 0.76) | 0.81  (0.65, 0.90) |
| REM latency, min | 2.2  (-31.5, 35.9) | 9.8  (-68.5, 88.2) | 16.1  (-115.6, 147.7) | 0.62  (0.540) | 1.01  (0.326) | 1.39  (0.173) | 0.94  (0.87, 0.97) | 0.81  (0.55, 0.93) | 0.71  (0.50, 0.85) |
| Arousal index | -6.0  (-17.1, 5.1) | -5.8  (-16.7, 5.1) | -3.5  (-22.3, 15.4) | -5.32  (<0.001) | -4.31  (<0.001) | -2.11  (0.042) | 0.82  (0.63, 0.92) | 0.72  (0.37, 0.89) | 0.68  (0.44, 0.82) |
| AHI and REI, index | -3.5  (-11.0, 4.1) | -10.2  (-17.2, -3.3) | -10.6  (-37.6, 16.5) | -4.31  (<0.001) | -11.49  (<0.001) | -4.34  (<0.001) | 0.58  (0.23, 0.80) | 0.62  (0.20, 0.85) | 0.72  (0.50, 0.85) |

note: WASO, wake after sleep onset; REM, rapid-eye-movement sleep; N, non-REM sleep, ICC, Intraclass correlation coefficient; Apnea-hypopnea index, AHI; REI, REI depending on SpO2; AHI measure by type I PSG, and REI measured by portable EEG device with SpO2.

**Supplementary Figure S1.** Image of InSomnograf K2®

**
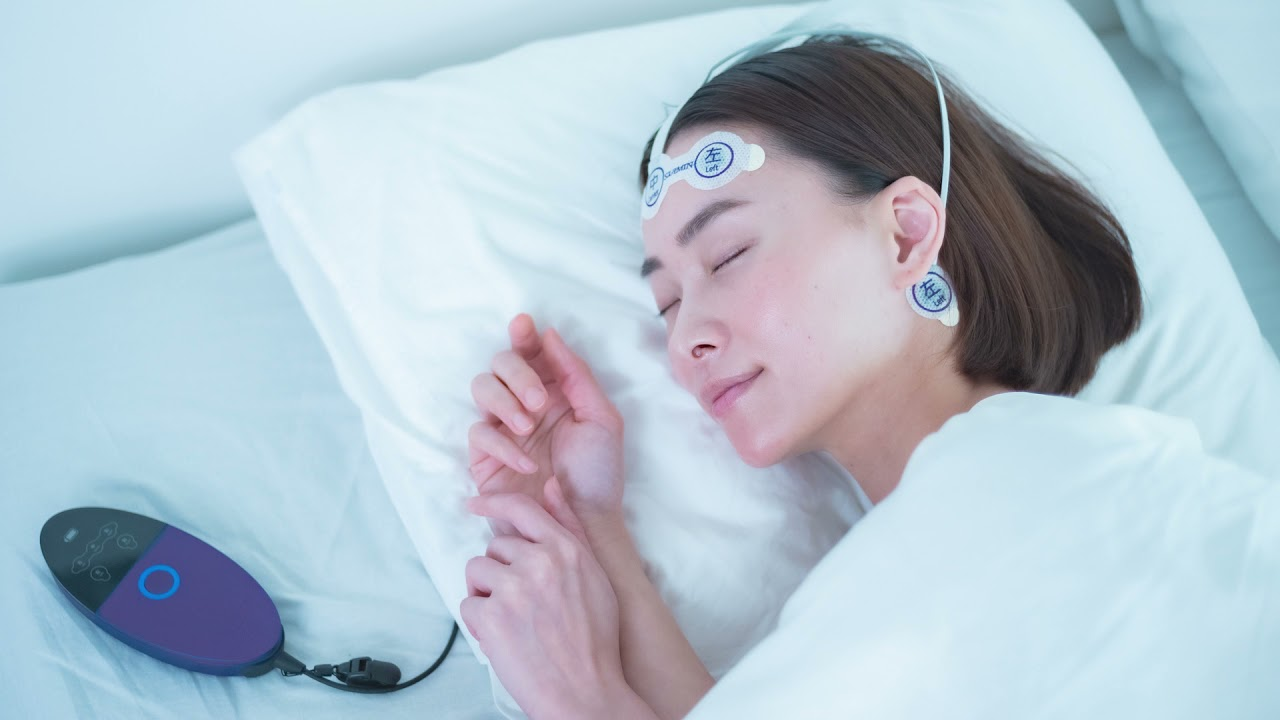
**

Publicly available at <https://www.suimin.co.jp/>

**Supplementary Figure S2.** Confusion matrix for sleep stage classification in all epochs of all recordings by OSA severity.

note: Parentheses indicate the percentage of agreement between the two devices. PSG, polysomnography, REM, rapid-eye-movement sleep; N, non-REM sleep.

**Supplementary Figure S3.** Comprehensive Analysis of Type I PSG and Portable EEG Devices by Epoch by OSA severity
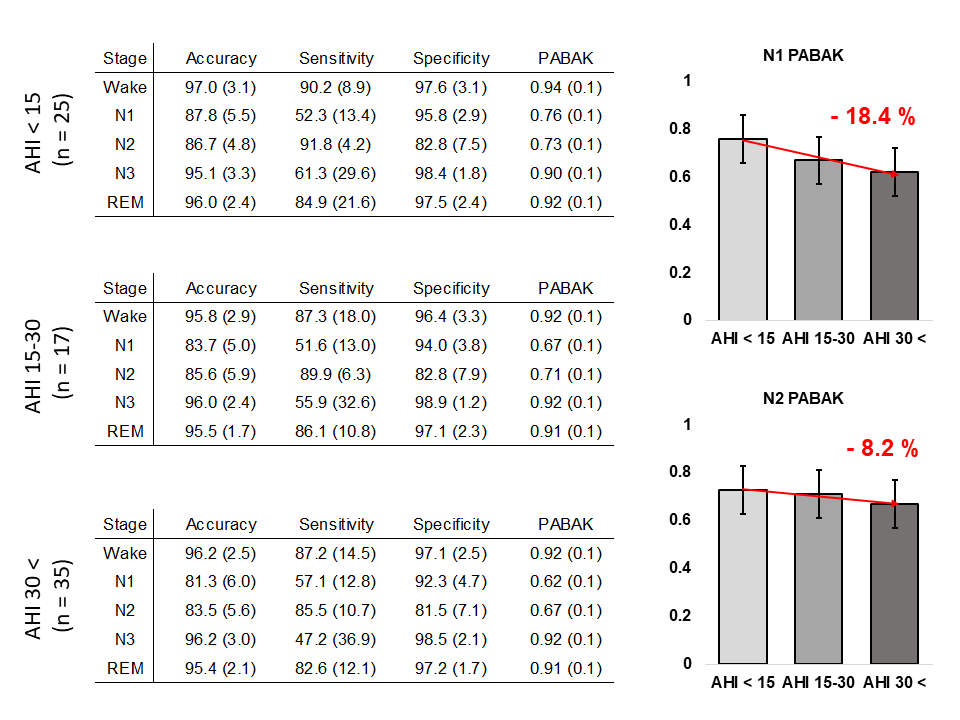


note: Parentheses indicate the standard error. PSG, polysomnography, REM, rapid-eye-movement sleep; N, non-REM sleep; PABAK, prevalence and bias adjusted Kappa.
